# Anatomy and Connectivity of the Subthalamic Nucleus in Humans and Non-human Primates

**DOI:** 10.3389/fnana.2020.00013

**Published:** 2020-04-22

**Authors:** Aron Emmi, Angelo Antonini, Veronica Macchi, Andrea Porzionato, Raffaele De Caro

**Affiliations:** ^1^Institute of Human Anatomy, Department of Neuroscience, University of Padua, Padua, Italy; ^2^Parkinson and Movement Disorders Unit, Neurology Clinic, Department of Neuroscience, University of Padua, Padua, Italy

**Keywords:** subthalamic nucleus, Parkinson, anatomy, connectivity, topography

## Abstract

The Subthalamic Nucleus (STh) is an oval-shaped diencephalic structure located ventrally to the thalamus, playing a fundamental role in the circuitry of the basal ganglia. In addition to being involved in the pathophysiology of several neurodegenerative disorders, such as Huntington’s and Parkinson’s disease, the STh is one of the target nuclei for deep brain stimulation. However, most of the anatomical evidence available derives from non-human primate studies. In this review, we will present the topographical and morphological organization of the nucleus and its connections to structurally and functionally related regions of the basal ganglia circuitry. We will also highlight the importance of additional research in humans focused on validating STh connectivity, cytoarchitectural organization, and its functional subdivision.

## Introduction

The Subthalamic Nucleus (STh)^1^, also known as Corpus Luysii, is an oval-shaped diencephalic structure located ventrally to the thalamus (Allheid et al., 1990; Parent and Hazrati, 1995; Joel and Weiner, 1997), playing a fundamental role in the circuitry of the basal ganglia. Described for the first time by Jules Bernard Luys (1865) (1828–1897), its functions remained largely unknown until 1927, when J. P. Martin reported the first case of hemichorea following lesion of the STh (Martin, 1927). As a key structure involved in the pathophysiology of several neurological disorders (e.g., Fedio et al., 1979; Martin and Gusella, 1986; Reiner et al., 1988; Albin et al., 1989b, 1990a, 1990b; Alexander et al., 1990; Bahatia and Marsden, 1994; Ferrante et al., 1994; Lozano et al., 2002; Temel et al., 2006a; Temel and Visser-Vandewalle, 2006; Hardman et al., 1997), the STh has also become a target for deep brain stimulation (DBS) in Parkinson’s Disease (PD) to modulate its firing patterns and improve clinical manifestations (Ranck, 1975; Lozano et al., 2002). In PD, the progressive loss of dopaminergic neurons in the substantia nigra pars compacta causes an alteration of the striatopallidal and pallidosubthalamical pathways in the basal ganglia, resulting in an abnormal burst-firing pattern in STh neurons (Magill et al., 2000; Urbain et al., 2002; Temel et al., 2005). DBS modulates STh firing, resulting in improvement in motor and non-motor disability as well as quality of life (Grill and Mcintyre, 2001; Dostrovsky and Lozano, 2002; Grill et al., 2004; Mcintyre et al., 2004). However, adverse side effects of the treatment are not to be underestimated (Saint-Cyr et al., 2000; Berney et al., 2002; Kulisevsky et al., 2002; Temel et al., 2006a, b): in a recent review Temel et al. (2006a, b) reported that out of 1389 patients who underwent bilateral STh DBS, 41% presented cognitive dysfunctions and decline in executive functions, 8% exhibited major depression symptoms and further 4% showed signs of hypomania. Even though the authors evidenced no association between the surgical approach to STh DBS and the side effects of the treatment, a possible explanation for cognitive and affective dysfunctions following DBS may reside in the functional subdivision of the STh, which is known to project to different circuits of the basal ganglia in primates (Carpenter et al., 1981b; Smith et al., 1990; Shink et al., 1996; Joel and Weiner, 1997). Thus, the stimulation of specific areas of the nucleus may modulate connectivity within associative and limbic circuits of the basal ganglia (Vergani et al., 2007; Dafsari et al., 2016, 2018a,b; Fabbri et al., 2017; Hamel et al., 2017; Antonini and Obeso, 2018; Petry-Schmelzer et al., 2019). In the following review we critically assess the available literature on STh morphology, morphometry and structural connectivity. With respect to other reviews in literature, we present a detailed description of the topographical organization of STh connectivity by comparing non-human primate tracing studies and human fiber tracking studies, evidencing similarities and differences between humans and non-human primates and methodological approaches used. The evolution of STh functional subdivision models is also briefly assessed and compared to progress achieved in human and non-human primate connectivity studies. Hence, our aim is to summarize the available anatomical evidence regarding connections and functional subdivisions of the STh, while suggesting aspects that require further investigation.

## Anatomy of the Subthalamic Nucleus

**FIGURE 1:**
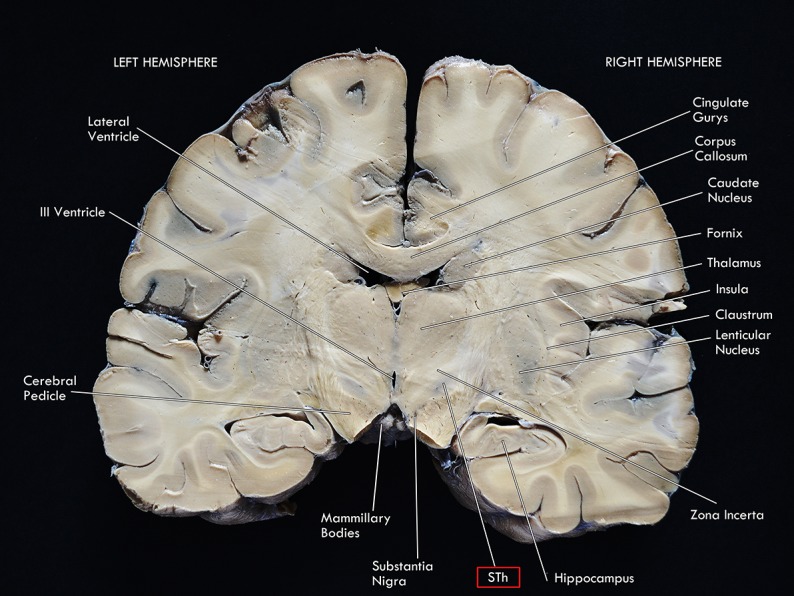
Coronal macrosection of the human brain at the level of the mammillary bodies. Human specimen from the body donation program of the Institute of Human Anatomy of the University of Padua. The STh (Subthalamic Nucleus) appears as the slightly dark area dorsal to the Substantia Nigra and ventral to the Zona Incerta.

**FIGURE 2:**
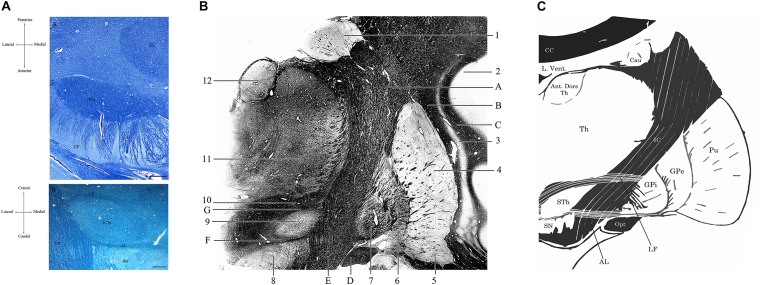
**(A)** Horizontal section of the human brain at the level of the mesencephalon (A). Klüver-Barrera Stain. 1.25× Magnification, scale bar = 500 μm. STh, subthalamic nucleus; CP, cerebral peduncle; RN, Red Nucleus. (B) Coronal section of the brain passing through the Subthalamic Nucleus, posteriorly to the anterior commissure. Luxol Fast Blue Stain. 1.25× Magnification, scale bar = 500 μm. STh, subthalamic nucleus; CP, cerebral peduncle; AL, ansa lenticularis; LF, lenticular fasciculus; SN, substantia nigra; ZI, zona incerta. Human specimen from the body donation program of the Institute of Human Anatomy of the University of Padua. **(B)** Coronal section passing through the postcommissural basal ganglia and the subthalamic nucleus, Weigert-Pal Stain. Human specimen from the body donation program of the Institute of Human Anatomy of the University of Padua. 1, Caudate nucleus; 2, Insular cortex; 3, Putamen; 4, Claustrum; 5, Ventral regions of the putamen, in relationship with the substantia innominata; 6, External Globus Pallidus; 7, Internal Globus Pallidus; 8, Substantia Nigra; 9, Subthalamic Nucleus; 10, Zona Incerta; 11, Lateral and medial thalamic nuclei; 12, Anterior dorsal nucleus of the thalamus. A, Internal capsule; B, External capsule; C, Extreme Capsule; D, Optic tract; E, Cerebral peduncle; F, Ansa lenticularis; G, Lenticular Fasciculus. **(C)** Digital schematization of a coronal section passing through the postcommissural basal ganglia, similar to the section seen in panel **(B)**. STh, Subthalamic Nucleus; SN, Substantia Nigra; GPi, Internal Globus Pallidus. GPe, External Globus Pallidus; Pu, Putamen; Th, thalamus; Ant. Dors Th, Anterior Dorsal nuclei of the Thalamus; Cau, Caudate nucleus; L. Vent., Lateral Ventricle; CC, Corpus Callosum; IC, Internal Capsule; Opt, Optic Tract; LF, Lenticular Fasciculus; AL, Ansa Lenticularis.

As a diencephalic structure, the STh has a close topographical relationship with the nuclei of the basal ganglia and with the structures of the mesencephalon (Figures 1, 2A). Dorsally, the STh confines with the zona incerta (ZI); the Field H2 of Forel, in particular the lenticular fasciculus, defines the dorsolateral margin of the nucleus, while the Field H1 of Forel, constituted mainly by the thalamic fasciculus, delineates part of its dorsomedial margin. The latter originates from the conjunction of the ansa lenticularis and part of the adjoining fibers of the lenticular fasciculus (Williams and Warwick, 1980), which separates the STh from the nucleus of the zona incerta. The ventromedial margin of the STh is delimitated by the ansa lenticularis, a white matter bundle originating from the ventral aspect of the lenticular nucleus which then joins the lenticular fasciculus to form the thalamic fasciculus, circumnavigating the medial aspect of the STh, as seen in Figures 2B,C.

Ventrally, the rostral regions of the STh confine with the dorsomedial aspect of the internal capsule as it continues into the base of the cerebral peduncle in the mesencephalon, while the most caudal part of the STh is situated atop the rostral extent of the substantia nigra (Allheid et al., 1990), in particular the dorsolateral aspect of the pars reticulata (Figures 2B,C). Dopaminergic nigrostriatal fibers pass dorsomedially to the STh through the medial forebrain bundle, where they enter the Fields of Forel and ascend to the striatum (Parent et al., 2000).

The STh is characterized by rich iron deposits (Rutledge et al., 1987) which can be evidenced through Perl’s stain in histological sections (Massey and Yousry, 2010; Massey et al., 2012); furthermore, these deposits are known to accentuate in pathological conditions, such as PD (Kosta et al., 2006). The vascular supply of the STh derives mainly from the perforating branches of the anterior choroidal artery and the posterior communicating artery, originating from the internal carotid artery, and posteromedial choroidal arteries, which derive from the superior cerebellar artery, hence from the vertebrobasilar circulation (Hamani et al., 2004).

From a cytoarchitectonical perspective, the STh of rodents, non-human primates and humans is largely characterized by the presence of excitatory glutamatergic neurons (Type I Grey Neurons), even though a small population of γ-aminobutyric acid interneurons (Type II Grey Neurons) has been identified (Iwahori, 1987; Albin et al., 1989a; Parent and Hazrati, 1995; Shink et al., 1996; Clarke et al., 1997; Kearney and Albin, 2000; Wang et al., 2000; Tai et al., 2001; Levesque and Parent, 2005; Marani et al., 2008). In rodents Kita et al. (1983) evidenced two morphologically diverse subpopulations of projection neurons in the STh through horseradish peroxidase labeling:

1. Type I STh Neurons, characterized by (a) axon collaterals contacting STh neurons at a local level, and by (b) dense dendritic arborizations, especially in proximity of the perykarion.
2. Type II STh Neurons, which possess no axon collaterals at a local level and present fewer dendrites at a proximal level.

**FIGURE 3:**
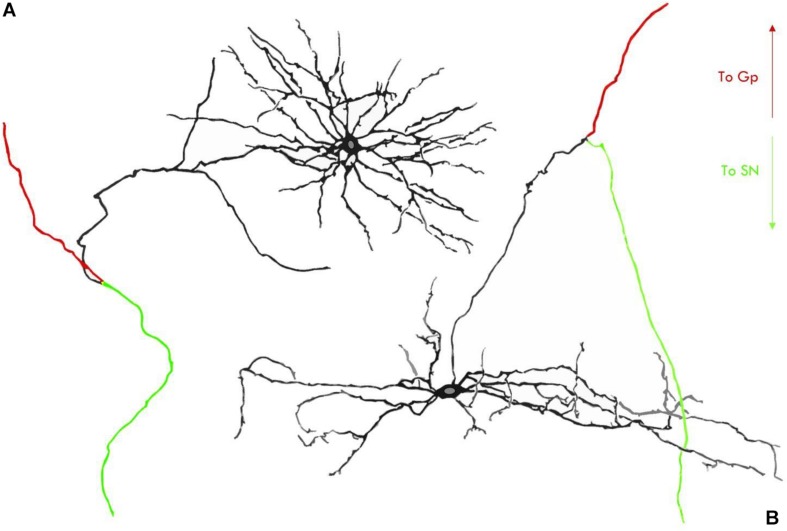
Neuron types in the STh according to rodent studies by Kita et al. (1983). **(A)** Type I STh neuron, characterized by local axon collaterals and dense proximal dendritic arborizations. **(B)** Type II STh neuron, with only two axon ramifications and less dense dendrites at a proximal level. GP, Globus Pallidus; SN, Substantia Nigra.

Both neuron types found in the rodent STh possess, however, a fundamental common feature which consists in a main axon dividing into two opposite-facing branches; the first branch ascends toward the lenticular nucleus, contacting mainly, but not exclusively, the globus pallidus; the second branch descends toward the mesencephalon and contacts the neurons of the substantia nigra pars compacta and pars reticulata (Gerfen et al., 1982; Kita et al., 1983; Kita and Kitai, 1987; Groenewegen and Berendse, 1990), as seen in Figure 3.

In primates, glutamatergic neurons in the STh are characterized by a large oval-shaped perykarion with a transverse diameter variable between 25 and 40 μm, whilst possessing wide dendritic arborizations which extend up to 400 μm from the soma; interneurons are more sparse, with fewer dendritic arborizations and generally smaller somata (Rafols and Fox, 1976; Yelnik and Percheron, 1979). Sato et al. (2000b) evidenced a branching pattern which varies in relationship to the target of the neurons: neurons contacting both the substantia nigra and the pallidal complex bifurcate in rostral and caudal branches similarly to the neurons identified in rodents by Kita et al. (1983), while neurons projecting only to the striatum or the pallidal complex present a single branch which bifurcates in proximity of its target; in particular, the authors identified five subtypes of subthalamic neurons in non-human primates based on their axon branching patterns: (1) neurons projecting to the SNr, GPi, and GPe (21.3%); (2) neurons projecting to the SNr and GPe (2.7%); (3) neurons projecting to the GPi and GPe (48%); (4) neurons projecting to the GPe (10.7%); (5) neurons projecting to the striatum (17.3%). It appears evident that, compared to rodents, non-human primates present a more diverse population of subthalamic neurons with different axon branching patterns. The typical axonal bifurcation encountered in rodents by Kita et al. (1983; Kita and Kitai, 1987) is present only in a small portion of primate STh neurons (type I and II STh neurons according to Sato et al. (2000b) accounting only for the 24% of the total population identified by Sato et al. (2000b). The remaining neurons present a branching pattern that has no direct correspondence in rodents.

Even though the morphology of subthalamic neurons has been adequately studied in rodents, primates, and even in minipigs (Larsen et al., 2004), only few studies have examined human specimen (Levesque and Parent, 2005), thus representing a potential field for further investigations.

Levesque and Parent (2005) studied the morphological characteristics of GABAergic interneurons within the STh compared to the main population projection neurons. The interneurons were identified by a distinct glutamic acid decarboxylase (GAD) immunoreactivity and presented smaller somata than projection neurons (12 versus 24 μm) with thinner and less numerous dendrites. According to the authors, these neurons appear similar to the interneurons identified in non-human primates by Rafols and Fox (1976) through Golgi silver impregnation. Another interesting aspect identified by Levesque and Parent (2005) is the lack of parvalbumin (PV) and calretinin (CR) immunoreactivity of human GABAergic interneurons within the STh; in fact, only projection neurons appear to be immunoreactive for these calcium binding proteins. Furthermore PV + neurons appear more abundant dorsolaterally within the STh, while CR + neurons were predominant in the ventromedial STh.

Through stereology, Hardman et al. (2002) compared the neuronal population of the STh in rodents, non-human primates and humans. While the number of neurons was proportional to the cross-sectional area of the STh across species, the proportional amount of neurons expressing parvalbumin (a calcium binding protein) was significantly lower in rodents compared to non-human primates and humans. Considering that the amount of PV + neurons increases in primates compared to rodents (Hardman et al., 2002) and that primate STh neurons present different axonal branching patterns compared to rodents (Sato et al., 2000b), one point of further investigation could be related to the expression of calcium binding proteins, such as parvalbumin, in the morphologically different populations of neurons within the STh. Given that the expression of calcium binding proteins is related to different firing properties of neurons (Hardman et al., 2002), a combined morphological and molecular characterization of neuronal populations within the STh could improve our understanding of the physiology of STh’s efferences and how external afferences are processed.

Unlike non-human primates, the morphology of human STh neurons has not yet been investigated through silver impregnation techniques. Morphologically different classes of STh neurons could represent functional subpopulations within the structure, possibly connected to different basal ganglia and cortical circuits.

### Cytoarchitectonics: Further Investigation

The morphological characterization of Human STh represents one of the main areas which requires further investigation. In humans, technical limitations determined by silver impregnation techniques, such as the incomplete impregnation of fine structures like axons and smaller dendrites in formalin fixed tissue, do not allow for a complete visualization of axonal branching patterns. However, features such as somata size and shape, number of dendrites and dendritic branching pattern could help in the identification of morphologically distinct neuronal subpopulations in humans. Another point of interest is the topographical distribution of the morphologically defined neuronal subpopulations within the structure: for example, what is the dendritic branching pattern of neurons found in the ventromedial STh compared to neurons in the dorsolateral STh? Does the neuronal morphology reflect the type of afferences received? Is there a relationship between morphological characteristics of STh neurons and functional territories within the structure?

### Morphometry of the STh

Few authors have approached the human STh from a morphometrical perspective and through the aid of unbiased stereology (Lange et al., 1976; Hardman et al., 2002; Levesque and Parent, 2005; Salvesen et al., 2015; Zwirner et al., 2017). We have reported stereology based studies on subjects with no clinical or pathological evidence in Table 1. Studies focused exclusively on subjects with clinical or pathological features were excluded, while only the values of healthy controls were included in the case of comparative studies between clinical and non-clinical populations (Salvesen et al., 2015).

**TABLE 1:** Morphometric studies on the human STh employing unbiased stereology.

Author	Embedding/tissue processing	Number of subjects	Section thickness	Section interval	Number of sections	Stereological method	Number of neurons (10^3^)	Volume (mm^3^)	Neuronal density (10^3^/mm^3^)
Lange et al. (1976)	Paraffin	NS	NS	NS	NS	NS	286–306	134–144	–
Hardman et al. (2002)	Immersion in PFA, cryoprotected, cut with freezing microtome	5	50 μm	1/20	6	Optical fractionator	561 ± 30	240 ± 23	–
Levesque and Parent (2005)	Immersion in PFA, cryoprotected, cut with freezing microtome	5	55 μm	1/20	6–9	Optical disector	239.5 ± 31.9	174.5 ± 20.4	Anteroposterior axis: 1.14 (anterior); 1.39 (middle); 1.52 (posterior). Dorsoventral axis: 1.30 (dorsal); 1.36 (central); 1.44 (ventral). Mediolateral axis: 1.56 (medial); 1.25 (central); 1.39 (lateral).
Salvesen et al. (2015)	Paraffin	10	40 μm	1/4	NS	Optical disector	450 (SD not available)	114.4 (SD not available)	8.0 (SD not available)
Zwirner et al. (2017)	Paraffin	14	20 μm	1/10	NS	Optical fractionator	431 ± 72	131.58 ± 19.83	4.8 ± 1.32 (Dorsal) 8.0 ± 2.1 (Ventral) 6.3 ± 0.91 (Medial)

From a methodological aspect, it must be noted that the stereological design varied notably across studies. Some authors (Hardman et al., 2002; Levesque and Parent, 2005) performed counting on 50–55 μm thick sections sampled at low ratio (1/20) with approximately 6–9 total sections evaluated, while other authors performed counting on 20 μm-thick paraffin-embedded sections sampled at higher ratio (1/10) (Zwirner et al., 2017). The age of the subjects included in the studies was also variable within and across studies (range is reported when standard deviation is missing): Lange et al. (1976): 50 years (24–99 range); Hardman et al. (2002): 74 ± 10 years; Levesque and Parent (2005): 50 ± 22.5 years; Salvesen et al. (2015): 69 years (60–75 range); Zwirner et al. (2017) 82 ± 10 years.

Tissue processing was also variable: in the case of Hardman et al. (2002) and Levesque and Parent (2005) the specimen were fixed in paraformaldehyde (PFA), cryoprotected in sucrose enriched phosphate-buffered saline (PBS) and cut with a cryostat. Lange et al. (1976), Salvesen et al. (2015) and Zwirner et al. (2017) employed paraffin embedding following formalin fixation. Due to the extensive dehydration of specimen during paraffin-embedding, significant shrinkage occurs and must be accounted for when estimating the volume of structures (Porzionato et al., 2015). While Zwirner et al. (2017) explicitly described shrinkage correction for volume estimation, the other two authors did not explicitly mention it in the manuscript.

The number of estimated neurons and the neuronal density in the STh varied significantly among studies. In the case of Salvesen et al. (2015), the values reported (900 × 10^3^) refer to the bilateral number of neurons obtained by multiplying by two the estimated number of neurons; hence, the mean unilateral value corresponds to 450 × 10^3^, and was reported as such in Table 1 (no standard deviation or variance was reported). The studies by Zwirner et al. (2017) and Salvesen et al. (2015) were conducted on more subjects compared to previous studies (14 versus 10, respectively) and reported more concordant values for the estimated amount of neurons (431 ± 72 × 10^3^ versus 450 × 10^3^, respectively); also, as stated by Zwirner et al. (2017) their reported morphometrical parameters also appeared to be concordant with Hardman et al. (2002) (561 ± 30), but not with Levesque and Parent (2005) (239.5 ± 31.9). This could be related to tissue processing, subject variability and age.

According to Levesque and Parent (2005), the neuronal density of the STh displays a decreasing gradient from the posterior to the anterior aspect of the nucleus, whilst displaying an increasing gradient from the dorsal to the ventral part of the structure; this is also confirmed by Zwirner et al. (2017). Furthermore, the authors evidenced how the ventral aspect, and in particular the medial and posterior thirds of the nucleus, displayed the highest neuronal density of the entire structure. This appears to be coherent with the tripartite subdivision of the STh, as a higher density of projection neurons and interneurons is a typical feature of associative/limbic areas and is thought to reflect the complex integrative activity of these regions (Wu and Parent, 2000; Levesque and Parent, 2005), thus supporting the role of ventral (medial and lateral) aspects of the nucleus in associative/limbic circuitry.

As for the volume, three out of five studies found in literature displayed similar values (ranging from 114 to 144 mm^3^), while the remaining two displayed higher values, ranging from 174 to 240 mm^3^ (Hardman et al., 2002; Levesque and Parent, 2005). As previously stated, the difference may be attributable to the employment of formalin fixation and paraffin embedding by the three studies reporting lower values, even though shrinkage correction was applied in most cases. Zwirner et al. (2017) combined post-mortem 3 Tesla MRI volume estimation with unbiased stereology, with highly consistent results. To date, this is the only study which has compared STh volumes through both methods.

### Morphometry: Further Investigation

Ultra-high field MRI should be employed in conjunction with unbiased stereology to accurately estimate STh volume. Gender and age related variations in volume, neuronal population and neuron density should also be taken into consideration.

## Subthalamic Nucleus Connectivity: Functional Subdivisions of Basal Ganglia Circuitry and Methods of Investigation

According to literature, the STh is characterized by several functional and regional subdivisions, with each region of the STh being structurally connected to either the sensomotor, cognitive or limbic circuit of the basal ganglia (Kita and Kitai, 1987; Smith et al., 1990; Parent and Hazrati, 1995; Joel and Weiner, 1997, 2000; Temel et al., 2006a). While several subdivisions of the basal ganglia have been proposed (Albin et al., 1989b), the most influential model of the organization of these structures views them as components of circuits connecting distinct thalamic, cortical and subcortical areas in a parallel manner, giving rise to relatively segregated connections, both anatomically and functionally (Alexander et al., 1986, 1990; Parent and Hazrati, 1995; Joel and Weiner, 1997, 2000). More recently, however, the structural segregation of the circuits of the basal ganglia has been questioned by several authors (Percheron et al., 1987, 1994; Reiner et al., 1988; Percheron and Fillon, 1991), providing evidence on the convergence of different afferent projections on dendritic fields in neurons of these subcortical structures (Smith et al., 1990, 1995; Shink et al., 1996). According to the classical tripartite organization proposed by Alexander et al. (1986), the basal ganglia can be subdivided into a motor and oculomotor circuit (forming together the general sensory-motor circuit of the basal ganglia); a dorsolateral prefrontal and lateral orbitofrontal circuit (forming the associative circuit); and an anterior cingulate circuit (also known as the limbic circuit).

• The motor circuit comprises mainly the primary, supplementary and pre-motor cortex; the dorsolateral part of the caudal putamen (postcommissural) and of the head of the caudate nucleus; the ventrolateral two thirds of the pallidal complex and part of the lateral substantia nigra; the ventrolateral, vental anterior and centromedian nucleus of the thalamus.
• The associative circuit comprises mainly the dorsolateral and ventrolateral prefrontal cortex; the rostral regions of the striatum (precommissural); the dorsomedial regions of the pallidal complex and most of the substantia nigra; the parvocellular part of the dorsomedial nucleus of the thalamus.
• The limbic circuit comprises the orbitofrontal cortical regions and the anterior cingulate; the nucleus accumbens and the ventral pallidum; the rostral External Globus Pallidus (GPe) and the rostral ventromedial Internal Globus Pallidus (GPi); the medial regions of the substantia nigra and the ventral tegmental area of the mesencephalon; the parvocellular part of the ventral anterior nucleus and the magnocellular part of the dorsomedial nucleus of the thalamus.

### Connectivity and Functional Subdivision of Subcortical Structures: Methods of Investigation

The connectivity and functional subdivision of nervous structures can be effectively studied through different means of investigation.

In non-human primates, the elective method to study the connectivity of subcortical structures such as the basal ganglia and the STh is represented by retrograde, anterograde or bidirectional tracing (Carpenter et al., 1981b; Smith et al., 1990; Joel and Weiner, 1997, 2000; Haynes and Haber, 2013; Coudé et al., 2018) and autoradiography (Nauta and Cole, 1978). Axonal tracing is currently considered the gold standard for studying anatomical connectivity in non-human primates. Through iontophoretic injection, bidirectional or anterograde tracers such as PHA-L (phaseolus vulgaris leucoagglutinin) and biotinylated dextran amine (Coudé et al., 2018) or retrograde tracers such as Fluoro-Gold (FG) and cholera toxin B (CTb) are injected in targeting sites through stereotaxic surgery, whilst animals are allowed to survive between 48 h and 14 days post-injection. The brains are then removed from the subjects and either postfixed in order to be processed for immunohistochemistry, or processed for autoradiography or confocal imaging; other tracing methods, such as the Marchi method (Whittier and Mettler, 1949) and silver techniques (Knook, 1965) were particularly popular in earlier studies, but are rarely found in recent literature.

In humans, structural and diffusion imaging techniques represent an important non-invasive tool for investigating STh connectivity both *in vivo* and *ex vivo*, even though important limitations must be considered. As far as DWI is concerned, the anatomical accuracy of brain connections derived from diffusion MRI tractography appears to be inherently limited (Jones et al., 2013; Thomas et al., 2014). According to Thomas et al. (2014) the anatomical accuracy of fiber reconstructions deriving from diffusion MRI appears to be highly dependent upon parameters of the tractography algorithm, with different optimal values for mapping different pathways. Tractography presents an inherent limitation in determining long-range anatomical projections based on voxel-averaged estimates of local fiber orientation obtained from DWI data. Furthermore, directionality of fibers can not be inferred, while the values regarding the connection strength between structures must be interpreted with care.

According to numerous authors (e.g., Jones et al., 2013; Thomas et al., 2014; Petersen et al., 2019) it appears necessary to complement tractography with histological and electrophysiological data in order to accurately map structural connectivity. In the case of STh research, MRI derived tractography should be based on the anatomical evidence derived by non-human primate tracing studies, as the electrophysiological properties of the STh can be studied effectively and accurately only through recordings in DBS patients, given the subcortical localization of the structure.

However, while electrophysiological recordings in DBS patients have greatly improved our understanding of the STh’s electrophysiological properties, measurements derive from an invasive approach requiring neurosurgery that allows recording only from patients with medical need for DBS (Alkemade et al., 2015). Hence, translation to normal STh physiology appears to be difficult, as it can be investigated in humans only in pathological states. Furthermore, the anatomical accuracy of these methods can not be compared to non-human primate fiber tracing and human MRI derived fiber tracking; while compelling results were provided by some authors (Fogelson et al., 2006) suggesting functional sub-loops between the STh and the motor areas of the cortex, the spatial resolution was too low to establish whether or not the recordings actually derived from the subthalamic area.

Considering the advantages and limitations of the aforementioned methods of investigation, anatomical evidence deriving from different sources should be integrated in order to generate an accurate representation of axonal pathways arising from and directed to the human STh.

Recently, Petersen et al. (2019) developed an holographic interface that allows the reconstruction of axonal pathways within the human brain by integrating different anatomical data deriving both from humans and non-human primates. In particular, the authors focused on the subthalamic region, with particular regard to STh connectivity. Data deriving from the vast array of non-human primate axon tracing studies was implemented within the interface and integrated with human histological and structural MRI studies. The holographic nature of the developed interface allowed for shared group sessions in which expert neuroanatomists reconstructed axonal pathways within the human subthalamic region basing on multifaceted anatomical evidence. According to the authors, the generated pathway atlas integrates anatomical detail regarding the STh in a manner that exceeds by far the capabilities of currently available tractography algorithms.

While the construction of the pathway atlas by Petersen et al. (2019) represents an important advance in modeling STh connectivity in humans, with important educational, clinical and research applications, limitations must also be considered. In fact, most connections reconstructed in the human pathway atlas derived from non-human primate studies. According to the authors, this requires inter-species assumptions on (a) the exact origin and termination points of fibers and (b) the maintenance of the somatotopic organization of the projections. Hence, non-human primate data should be integrated with other sources of anatomical evidence in humans, such as ultra-high field MRI (7 Tesla), to further enhance the accuracy of reconstructions.

### STh Functional Subdivision: Tripartite Hypothesis

As a fundamental part of the basal ganglia circuitry, the STh is also connected to the circuits of the basal ganglia and presents, according to classical literature, a relatively distinct functional subdivision. The subdivision of the STh is founded on the connections which arise from the different brain regions conjoining with the aforementioned functional circuits. Thus, a peculiar region of the STh is defined mainly by the connections it forms with other cortical and subcortical structures and their relationship with the functional circuitry of the basal ganglia, while its intrinsic cytoarchitecture remains a current topic of investigation.

**FIGURE 4:**
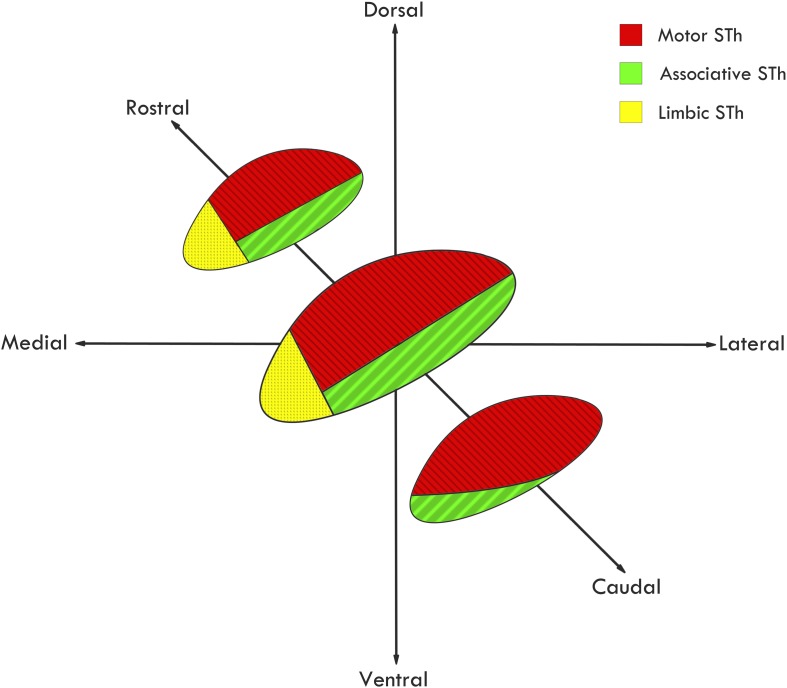
Functional subdivision of the STh according to the classical tripartite hypothesis. Redrawn from various sources (Parent and Hazrati, 1995; Hamani et al., 2004).

Among the very first to extensively review STh connectivity in non-human primates, although without considering its functional subdivision, Whittier and Mettler (1949) evidenced that out of 44 authors, 22 reported connections between the STh and the globus pallidus, while fewer reported projections to other areas, such as the mesencephalic tegmentum, putamen, substantia nigra, caudate nucleus, red nucleus, thalamus and commissural connections with the controlateral STh. As for afferent projections, extensive input appeared to originate mainly from the GPe (Ranson and Berry, 1941; Ranson et al., 1941; Nauta and Mehler, 1966). Successive studies further confirmed the importance of pallido-subthalamic projections, whilst expanding the STh circuitry by evidencing cortico-subthalamic, reticulo-subthalamic and thalamo-subthalamic afferences (Nauta and Cole, 1978; Smith et al., 1990; Parent and Hazrati, 1995; Shink et al., 1996). Basing on non-human primate tracing studies, a functional subdivision of the nucleus has been proposed by Parent and Hazrati (1995) and expanded by Joel and Weiner (1997): according to the authors, the medial third of the rostral two-thirds of the STh is connected mainly, but not exclusively, to the limbic circuit; the dorsolateral aspect of the rostral two thirds and most of the caudal third of the STh is connected mainly with the motor circuit; the ventrolateral aspect of the rostral two thirds and a small ventromedial region of the caudal third of the STh is connected with the associative circuit, as seen in Figure 4. This gives rise to a functional tripartition of the structure, which represents the main trend in classical literature on STh regionalization and connectivity, and has layed the ground for investigations on STh functional subdivision and connectivity in humans.

As for STh connectivity in humans, Aravamuthan et al. (2007) were among the first authors to employ diffusion MRI (1.5 Tesla) to study STh connectivity in living subjects through probabilistic tractography. The authors identified several regions with high probability of connection to the STh; these included the dorsal premotor cortex, the supplementary motor area, and the primary motor area for the lower limbs, trunk, arm and forearm, while the probable subcortical connections of the STh included the thalamus, pallidal complex, substantia nigra, and pedunculopontine nucleus. A topographical organization of the STh was also reported, with the motor cortical regions being connected to the dorsal STh and the associative cortical regions being connected to the inferior and medial STh. It must be noted, however, that the authors defined a single voxel based on surrounding landmarks that they were confident fell within the STh (Lambert et al., 2012); hence, results must be considered with caution, as the STh was not actually anatomically identified.

On the other hand, Lambert et al. (2012) were the first to employ a data driven method with DWI (in living human subjects) that evidenced three distinct clusters within the STh based on brain connectivity profiles, largely corresponding to the functional tripartition hypothesized in non-human primates. The authors sub-parcellated the STh into the aforementioned functional territories and evidenced connections between the STh and subcortical structures that were coherent with the tripartite hypothesis; most cortical regions, on the other side, possessed some connections to all of the defined STh territories.

• The posterior aspect of the STh was identified as the motor region and showed connections with the posterior insula, posterior putamen and GPe, mid-caudate and ventro-lateral thalamic nuclei.
• The anterior STh was defined as the limbic region and was connected to the baso-lateral amygdala, mid-inferior putamen, mid-GPe and ventral-anterior thalamus.
• The associative STh was defined as a region with connections to both limbic and motor circuits which represented more of a functional gradient between motor and limbic territories rather than a distinct subregion.

It must be noted that, even though Lambert et al.’s (2012) study seems to support the tripartite hypothesis basing on the STh subparcellization, the authors clearly state that the STh territory commonly defined as associative actually represents an overlapping and topographically arranged transition between the limbic and motor territories, thus supporting the idea of functionally overlapping regions within the STh (Lambert et al., 2015).

Probabilistic tractography, employed by both groups albeit with significantly different methodologies, presents inherent limitations, as it estimates the probability of connection between seed and target regions. As stated by Lambert et al. (2012), seed and target region size, distance of tracking, regions with dense crossing fibers, MRI artifact and noise are all factors which can greatly influence fiber tracking. Again, results deriving from tractography must be interpreted with care and require complementary validation by histology and non-human primate studies.

### Challenges to the Tripartite Hypothesis

A recent review by Keuken et al. (2012) has evidenced how variability across non-human primate tracing studies is not to be underestimated, with many authors disagreeing on both the number of STh subdivisions and on the localization of such subregions. Keuken et al. (2012) also advance the hypothesis of more than three functional subdivisions within the STh, with regard to Alexander et al. (1990) aforementioned hypothesis of five parallel loop circuits within the basal ganglia (sensorimotor, oculomotor, dorsolateral prefrontal, orbitofrontal, and anterior cingulate circuits).

Another critical point in the classical tripartite hypothesis of the STh is the segregation of anatomo-functional circuits; while earlier studies suggested relatively segregated and defined STh subdivisions, more recent evidence points toward overlapping subregions and converging axonal afferents on large dendritic fields of neuronal populations of the structure (Percheron et al., 1987, 1994; Reiner et al., 1988; Smith et al., 1990, 1995; Percheron and Fillon, 1991,; Shink et al., 1996; Haynes and Haber, 2013; Alkemade et al., 2015).

As summarized by Alkemade and Forstmann (2014), evidence points toward a topographical organization of the STh without defined anatomical borders, with at least partial overlap between functional subregions within the structure; furthermore, according to the authors, the extent of functional overlap within STh subregions is still unclear and represents a point of further investigation.

On the other hand, numerous tracing studies aimed to identify the connections between the STh and other brain structures without considering their intrinsic functional subdivision. In fact, distribution of tracers is related to (a) injection site and (b) diffusion of the tracer within the injected structure; numerous studies, especially early tracing studies prior to the 1980s, did not consider the functional subdivision of the injection sites (Kim et al., 1976; Nauta and Cole, 1978; Smith and Parent, 1986; Parent and Smith, 1987). This is also related to the difficulty of selectively injecting small brain structures and to the intrinsic diffusion of the tracer within the site, along with the issue related to tracer diffusion to unrelated fibers passing within or close by large injection site. Furthermore, other studies only considered the connection between specific structures (such as the brainstem nuclei) and the STh (Lavoie and Parent, 1994c; Rico et al., 2010), which may or may not be related to the functional subdivision of the basal ganglia. Confirmation for the classical tripartite hypothesis has been recently provided by the very large tracing study carried out by Haynes and Haber (2013), indicating topographically organized projections arising from cortical areas directed to specific regions within the STh compatible with the subdivision proposed by previous authors (Parent and Hazrati, 1995; Joel and Weiner, 1997).

In conclusion, controversy on STh subdivision location, boundaries and circuit segregation remains a topic of current debate between research groups, and further confirmation is needed by combining ultra-high field MRI, histology and cytoarchitectonics (Alkemade et al., 2015).

In the following paragraphs, the connectivity of the STh will be studied considering mainly non-human primate tracing studies, whilst also integrating information coming from the few available human fiber tracking studies through DTI and DWI both *in vivo* and *ex vivo*, in order to highlight aspects which require further investigation in humans. For an extensive revision of non-human primate *in vivo* tracing studies involving the STh, refer to Table 2.

**TABLE 2:** Non-human primate axon tracing studies.

Author(s)	Species	Technique	Tracing agent	PI.T.*	Injection site(s)	Connection(s) evidenced
Carpenter et al. (1976)	*Rhesus monkeys*	Anterograde tracing (Autoradiography)	[^3^H]- aminoacids (L-leucine, L-proline, L-lysine)	68 h–22 days	Rostrolateral SN	Whole extent of the STh
					Caudolateral SN	No connections to STh evidenced
					Rostromedial SN	Medial STh
					Central SN	No connections to STh evidenced
Kim et al. (1976)	*Rhesus monkeys*	Anterograde tracing (Autoradiography)	[^3^H]- aminoacids (L-leucine, L-proline, L-lysine)	5–28 days	GPi	No connections to STh evidenced
					GPe	Whole STh except caudal and medial regions
					Putamen	No connections to STh evidenced
Kunzle and Akert (1977)	*Macaca fascicularis*	Anterograde tracing (Autoradiography)	^3^H-proline	2–3 days	Brodmann area 8	Subthalamic nucleus (no topography specified)
Nauta and Cole (1978)	*Macaca mulatta*	Anterograde tracing (Autoradiography)	Tritiated aminoacids (NS)	7–13 days	STh	1. Pallidal complex 2. SN 3. Ventral lateral and ventral anterior thalamic nuclei 4. PPT (pars compacta) 5. Putamen
Von Monakow et al. (1978)	*Macaca fascicularis*	Anterograde tracing (Autoradiography)	Radioactive labels: arginine, leucine and proline.	2–8 days	Brodmann area 4	Dorsal and lateral STh
					Brodmann area 6	Central STh
					Brodmann area 8	Ventral STh
					Brodmann area 9	No connections to STh
					Brodmann area 3,1,2	No connections to STh
DeVito et al. (1980)	*Macaca mulatta*	Retrograde tracing	Horseradish peroxidase	48–65 h	GPi	Central and ventromedial STh
Carpenter et al. (1981a)	Squirrel monkey (*Samiri sciureus*)	Retrograde tracing	Horseradish peroxidase	24–72 h	Outer portion of the GPi (rostral and dorsal regions)	Ventral and medial thirds of the caudal third of the STh
					Central GPi	Medial third of the caudal third of the STh
					Apical GPi	Medial third of the caudal two thirds of the STh
					Rostral division of the GPe	Medial third of the middle third of the nulceus
					Central division of the GPe	Whole rostral third of the STh; central and dorsal regions of the caudal two-thirds of the STh
		Anterograde tracing (Autoradiography)	^3^H-aminoacids (NS)	NS	GPi	No connections evidenced to STh
					Rostral division of the GPe	Medial two-thirds of the rostral third of the STh; Central part of the middle third of the STh
					Central division of the GPe	Lateral third of the STh
Carpenter et al. (1981b)	Squirrel monkey (*Samiri sciureus*)	Retrograde tracing	Horseradish peroxidase	48 h	Subthalamic nucleus	1. GPe, regions adjacent to the lateral medullary lamina 2. SN (pars compacta and pars reticulata) 3. PPT 4. Dorsal nucleus of raphe
		Anterograde tracing (Autoradiography)	L-leucine + L-proline	5–8 days	Subthalamic nucleus	1. Pallidal complex (with inverse dorsoventral topography) 2. Substantia nigra 3. Ventral anterior, ventral lateral and dorsomedial nuclei of the thalamus
					Substantia Nigra	Subthalamic nucleus (no topography specified)
DeVito and Anderson (1982)	*Macaca Mulatta*	Anterograde tracing (Autoradiography)	L-proline + L-leucine + L-lysine	48–65 h	Internal Globus Pallidus	Uncertain connection to STh
					External Globus Pallidus	Whole STh
Smith and Parent (1986)	Squirrel monkey (*Samiri sciureus*)	Bidirectional tracing	Wheat germ agglutinin – horseradish peroxidase conjugate (WGA-HRP)	48 h	Putamen	Dorsolateral STh
					Caudate nucleus	Ventromedial STh
					Motor and premotor cortex	No connections evidenced to STh
Parent and Smith (1987)	Squirrel monkey (*Samiri sciureus*)	Anterograde tracing	∙ Wheat germ agglutinin – horseradish peroxidase conjugate (WGA-HRP) ∙ Nuclear yellow ∙ Fast blue	18–48 h	Putamen	Rostral dorsolateral two-thirds of the STh
					Caudate nucleus	Rostral ventromedial STh
					Substantia nigra	Ventromedial STh
					Globus pallidus	Whole STh, more evident in dorsolateral STh
					Pedunculopontine nucleus	Central regions of the STh
Smith et al. (1990)	Squirrel monkey (*Samiri sciureus*)	Anterograde tracing	Phaseolus vulgaris-leucoagglutinin	10–12 days	STh	1. Pallidal complex 2. Striatum (middle third of the putamen and caudate nucleus) 3. SN pars compacta 4. SN pars reticulata 5. Mesencephalic and pontine tegmentum (PPT and periacqueductal gray)
Sadikot et al. (1992)	Squirrel monkey (*Samiri sciureus*)	Anterograde tracing	Phaseolus vulgaris-leucoagglutinin	12–14 days	Centromedian nucleus	Dorsolateral STh
					Parafascicular nucleus	Medial and rostral STh
Hazrati and Parent (1992)	Squirrel monkey (*Samiri sciureus*)	Anterograde tracing	Phaseolus vulgaris-leucoagglutinin; Biocytin	2–12 days	Putamen	No connections investigated to the STh
					STh	Globus pallidus (no topography specified)
Lavoie and Parent (1994)	Squirrel monkey (*Samiri sciureus*)	Anterograde tracing	Phaseolus vulgaris-leucoagglutinin; [H]leucine	6–12 days	PPT	Whole extent of the STh
Shink et al. (1996)	Squirrel monkey (*Samiri sciureus*)	Anterograde tracing	Biotin dextran amine	7–10 days	Pallidal complex (both segments) – different injection sites	Inverse dorsoventral topography of STh connections (i.e., dorsal pallidal regions are connected with ventral STh regions, and vice-versa)
					GPi – different injection sites	
			Phaseolus vulgaris-leucoagglutinin	7–10 days	GPe – different injection sites	
François et al. (2000)	*Cercopithecus aethiops*	Anterograde tracing	Biotin dextran amine	10 days	Peri- and retrorubral area (cathecolaminergic cell group A8)	Whole extent of the STh
					SN pars compacta (cathecolaminergic cell group A9)	Anteromedial STh
		Retrograde tracing	Fluoro-gold	10 days	STh	Whole mesencephalon
Sato et al. (2000a)	*Macaca fascicularis*	Anterograde tracing	Biotin dextran amine	48–72 h	GPe	Medial GPe is connected to Medial STh Dorsal GPe is connected to Ventral STh Ventral GPe is connected to Dorsal STh
Sato et al. (2000b)	*Macaca fascicularis*	Anterograde tracing	Biotin dextran amine	48–72 h	STh	1. SN pars reticulata 2. GPe 3. GPi 4. Striatum
Giolli et al. (2001)	*Macaca mulatta* and *Macaca fascicularis*	Anterograde tracing (Autoradiography)	[H]-leucine	5–7 days	Different cortical areas	No connections investigated to the STh
		Retrograde tracing	Wheat germ agglutinin – horseradish peroxidase conjugate (WGA-HRP)	48 h	Nucleus reticularis tegmenti pontis	STh (topography not specified)
Kelly and Strick (2004)	*Cebus Apella*	Retrograde tracing	Rabies virus	4 days	Primary motor area (M1)	3rd Order neurons within the dorsolateral STh
					Brodmann area 46	3rd Order neurons within the rostral ventromedial STh
Karachi et al., 2005	*Cercopithecus aethiops* and *Macaca mulatta* and *Macaca fascicularis*	Retrograde tracing	Wheat germ agglutinin – horseradish peroxidase conjugate (WGA-HRP)	3 days	Motor GPe Associative GPe Limbic GPe	Motor GPe is connected to posterior and dorsal regions of the STh Assocative GPe is connected to anterior central and posterior ventrolateral STh Limbic GPe is connected to the anterior medioventral STh
		Anterograde tracing	Biotin dextran amine	10 days	Motor GPe Associative GPe Limbic GPe	
Tandé et al. (2006)	*Cercopithecus aethiops* and *Macaca mulatta*	Retrograde tracing	Fluoro-gold	10 days	Subthalamic Nulceus	Parafascicular nulceus
Miyachi et al. (2006)	Macaque monkeys	Retrograde tracing	Rabies virus	4 days	Somatotopically defined regions of the primary motor cortex (M1)	Orofacial M1 is connected to ventrolateral STh; Hindlimb M1 is connected to dorsomedial STh; Forelimb M1 is connected to the two aforementioned STh regions.
Rico et al. (2010)	*Macaca fascicularis*	Retrograde tracing	Cholera toxin B	14 days	Ventral anterior and ventral lateral nuclei of the thalamus	Mainly medial STh
Bostan et al., 2010	*Cebus apella*	Retrograde Tracing	Rabies virus	42 h	Crus IIp and lobule VIIb of the cerebellum	Controlateral STh; Crus IIp presents more connections to the rostral STh and more ventromedially, while lobule VIIb presents more connections to the caudal STh
Haynes and Haber (2013)	Macaque Monkeys (*Macaca fascicularis* + *Macaca nemestrina*)	Anterograde + Bidirectional tracing	Lucifer Yellow, Fluororuby or Fluorescin coniugated with dextran amine or tritiated amino acids.	12–14 days	Orbitofrontal cortex/ventromedial prefrontal cortex	Uncertain (mostly outside the medial border of STh)
					Dorsal Anterior Cingulate Cortex	Medial tip of the STh
					Dorsal prefrontal cortex (B.A. 9–46)	Medial half of the STh
					Rostral dorsal prefrontal cortex (rostral B.A. 6)	Medial half of the STh, mainly caudal.
					Caudal dorsal prefrontal cortex (caudal B.A. 6)	Ventrolateral STh
					Primary motor cortex	Dorsolateral STh
Ishida et al. (2016)	*Macaca fuscata*	Retrograde tracing	Rabies virus	3–4 days	Ventral premotor cortex	Caudal dorsolateral STh and rostral ventrolateral STh
Coudé et al. (2018)	*Macaca fascicularis*	Anterograde tracing	Biotin dextran amine	8–10 days	Layer V of primary motor cortex	Dorsolateral STh

* PI.T.: Post injection time, i.e., period between tracer injection and sacrifice. STh, Subthalamic Nucleus; GPe, External Globus Pallidus; GPi, Internal Globus Pallidus; SN, Substantia Nigra; PPT, pedunculopontine tegmental nucleus; B.A., Brodmann area.

## STh Connections in Non-Human Primates and Humans

### Cortical Connections With the STh

The existence of cortico-subthalamic projections is well documented in non-human primates (Kunzle and Akert, 1977; Von Monakow et al., 1978; Parent and Hazrati, 1995; Haynes and Haber, 2013; Ishida et al., 2016; Coudé et al., 2018), with few studies investigating the pathway also in humans through DTI and DWI. This pathway is also referred to as the “hyperdirect pathway” of the basal ganglia, in regards to the direct and indirect pathway subdivision.

#### Non-human Primate Studies

The very first authors to report cortico-subthalamic projections were Kunzle and Akert (1977), evidencing diffuse projections to the STh originating from Brodmann area 8. Further investigation by Von Monakow et al. (1978) evidenced a peculiar topography of the connections arising from the frontal cortex: Brodmann area 4 appears to be connected with the dorsolateral regions of the STh; Brodmann area 6 appears to be connected to the central third of the STh, while Brodmann area 8 appears to be connected to the ventral regions of the STh. The authors were unable to identify any projections arising from Brodmann areas 9 and 3,1,2.

Recently, Haynes and Haber (2013) conducted an extensive study on the cortical projections to the STh in non-human primates, evidencing the following connections: the dorsal anterior cingulate cortex appears to be connected to the medial tip of the STh (limbic circuit); the dorsal prefrontal cortex (Brodmann area 9 and 46) projects to the medial half of the STh; the rostral dorsal prefrontal cortex (rostral Brodmann area 6) projects to the medial half of the caudal STh; the caudal dorsal prefrontal motor cortex (caudal Brodmann area 6) projects to the ventrolateral STh, while the primary motor cortex shows connections to the dorsolateral regions of the STh (thus part of the motor circuit). No clear connections between the ventromedial prefrontal cortex and the STh were evidenced. These results were further confirmed by Ishida et al. (2016) and Coudé et al. (2018) which revealed that the ventral premotor cortex is connected mainly to the caudal dorsolateral STh and rostral ventrolateral STh, and that the projections arising from the motor cortex originate from the V layer (deep pyramidal cell layer). Interestingly, Coudé et al. (2018) evidenced how the cortico-subthalamic fibers are actually axon collaterals of corticofugal fibers that in most cases also project to the zona incerta and the red nucleus. Hence, in accordance to these results, the hyperdirect pathway from the cortex is not exclusively devoted to the STh, but projects also to other subcortical nuclei.

With particular regard to the projections arising from the primary motor cortex (M1), a somatotopic organization is reported (Parent and Hazrati, 1995; Hamani et al., 2004): the leg, arm and orofacial structures appear to be represented in the medial, lateral and dorsolateral portions of the STh, respectively. According to Nambu et al. (1996, 1997, 2000) the lateral portion of the STh receives projections from the primary motor cortex and presents a medial to lateral representation of the leg, arm and face, while the medial portion of the nucleus receives fibers from the supplementary motor area, dorsal and ventral premotor cortex and presents an inverse somatotopic distribution. The somatotopic organization of M1 projections to STh has been confirmed in Miyachi et al.’s study (2006), in accordance to Nambu et al. (1996, 2000).

To date, no studies reported subthalamo-cortical projections.

From a cytoarchitectural and chemoarchitectural perspective, the cortico-subthalamic pathway utilizes glutamate as main neurotransmitter, and the terminals of the projections contact mainly small dendrites; in particular, projections arising from the motor cortex appear to contact mainly, but not exclusively, distal dendrites and other afferent axons directed toward the STh (Coudé et al., 2018).

#### Human Tractography Studies

Connectivity between the cortex and the STh has been investigated in humans using DTI and DWI in both controls and PD patients (Aravamuthan et al., 2007; Lambert et al., 2012; Petersen et al., 2017; Isaacs et al., 2018; Plantinga et al., 2018). The studies by Aravamuthan et al. (2007) and Lambert et al. (2012) were discussed previously and will be briefly summarized with regard to cortical connections to the STh. Through probabilistic tractography Aravamuthan et al. (2007) evidenced connections between the motor cortical areas and the dorsal STh, while associative regions of the cortex were connected to the inferior and medial STh. Lambert et al. (2012), on the other hand, evidenced how most cortical regions possessed at least some connections to all of the functional territories identified within the STh, without a precise topographical organization. Petersen et al. (2017) investigated cortico-subthalamic projections in PD patients and controls, using both probabilistic and deterministic tractography; projections to the STh derived mainly from the motor cortex, with particular regard to BA6 and BA4. The probabilistic method further identified a larger portion of projections connecting BA6 to the STh, compared to the projections from the BA4 to the STh. As for the target STh regions, the authors identified the dorsolateral aspect of the nucleus as the main site of projection of cortical fibers; this appears to be coherent with the tripartite hypothesis of the STh, where the dorsolateral aspect represents the motor subregion of the structure. Isaacs et al. (2018) studied the projections arising from the cortex and directed to the STh and the striatum by employing DWI and resting-state functional MRI. The cortical areas of interest were identified basing on primate tracing studies. Comparatively, the striatum appeared to receive more projections from the cortex when compared to the STh, with the exception of the orbitofrontal cortex and the ventromedial prefrontal cortex, whose tract strength were stronger for the STh. Both structures received strong projections from the cingulate motor area and from the supplementary motor area. The study did not assess the topographical organization of the projections, but rather focused on tract strengths between cortical areas and subcortical targets. However, several limitations to the estimation of connection strengths must be noted. As stated by the authors, the exact identification of white matter “entrance points” within the cortex appears particularly challenging, giving rise to what is generally referred as “gyral biases” (Isaacs et al., 2018). Furthermore, the term “tract strength” is referred to the amount of streamlines originating from the seed region (i.e., the cortex) and terminating on the target area (i.e., subcortical structures, including the STh), but does not quantify the actual number of white matter fibers, and its values are related to the size of the target structure. Hence, results related to tract strengths should not be over interpreted (Jones et al., 2013).

Plantinga et al. (2018) studied the connectivity between cortical areas and the STh with regards to the parcellation of the structure in PD patients using 7 Tesla MRI. The authors evidenced connections between the motor areas of the cortex (Primary motor, supplementary motor and premotor cortex) and the posterior dorsolateral aspect of the STh, the associative areas of the cortex and the central region of the STh, partially overlapping with the motor area, and the limbic areas of the cortex (hippocampus, amygdala, cingulate gyrus, orbitofrontal cortex) with the anterior ventromedial aspect of the STh; the authors also identified volumetric differences between the areas, with the 55.3 ± 14%, 55.6 ± 15.9% and 20.3 ± 16.3% of the total volume of the nucleus being occupied by the posterior dorsolateral motor region, central associative region and anterior ventromedial limbic region, respectively. This subdivision seems to confirm, along with the other human tracing studies, the classical tripartite hypothesis.

#### Future Perspectives

To date, the presence of cortico-subthalamic fibers has been evidenced in humans only through tractography. Histological studies present inherent technical limitations in reconstructing fiber pathways from the cortex to the STh due to the conspicuous length of the fibers. Within this context, post-mortem MRI-derived tractography could be employed to further characterize the connections between the cortex and the STh, similarly to Plantinga et al.’s (2016) study on STh connections to the GPe and SN. The higher resolution achievable through longer scan times could provide useful information on the characterization of connections between the cortex and the STh.

Another point of further investigation is related to the overlap between functional territories within the STh. As recent literature suggests (Alkemade and Forstmann, 2014; Lambert et al., 2015), the associative territories of the STh could represent a transition gradient between motor and limbic regions, rather than an anatomically distinct area with defined boundaries. The high resolution achieved through Ultra-High field MRI on *ex vivo* human specimen could shed more light on the precise topography of cortical connections with the STh.

### Pallidal (GPe and GPi) Connections With the STh

#### Subthalamo-Pallidal Projections

##### Non-human Primate Studies

Through autoradiography, Nauta and Cole (1978) confirmed the existence of a highly organized subthalamo-pallidal projection which, originating from the STh, traverses the internal capsule and ends on the pallidal complex (both segments) describing a route identical, but with opposite directionality, to the second division of the ansa lenticularis. In particular, the medial part of the rostral third of the STh projects to the rostral GPe and ventromedial GPi, including the ventral pallidum, while the dorsal regions of the caudal STh project toward the ventrolateral two thirds of the caudal pallidal complex. Furthermore, the authors were unable to confirm any commissural connection between the STh of each hemisphere. According to Carpenter et al. (1981a, b); Carpenter and Jayaraman (1990) projections from the STh possess a topographical organization. Subthalamo-pallidal (efferent) projections directed toward the GPe are organized as follows: the rostral third of the STh projects to the central part of the GPe; the central third of the STh projects to the rostral part of the GPe; the caudal third, especially the lateral surface, projects toward the caudal GPe. Subthalamo-pallidal (efferent) projections directed toward the GPi originate mainly from the medial third of the caudal STh. According to the authors, the rostral regions of the STh do not project to the GPi. A common characteristic of subthalamo-pallidal projections evidenced by both Nauta and Cole (1978) and Carpenter et al. Carpenter et al. (1981a, b); Carpenter and Jayaraman (1990) is the inverse dorsoventral topography of the fibers, i.e., the dorsal regions of the STh project to the ventral regions, while the ventral regions of the STh project to the dorsal regions of the pallidal complex. These findings were further confirmed and extended by Shink et al. (1996) and Smith et al. (1990, 1995), who also evidenced a convergence at a synaptic level: many STh neurons projecting to the GPi received synaptic input from GPe collaterals, whose neurons were originally directed to the same area of the GPi. Similarly, GPe neurons projecting to the GPi received input from axon collaterals of STh neurons mainly projecting to the same areas of the GPi.

**FIGURE 5:**
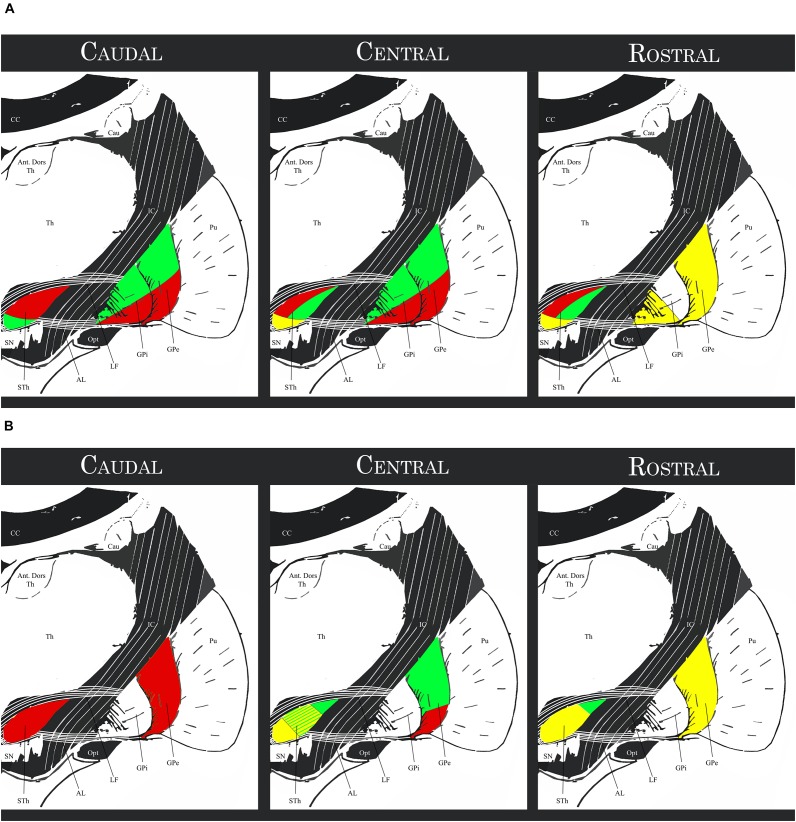
**(A)** Schematic topography of the Subthalamo-pallidal efferents. Red regions: motor circuits; Green regions: associative circuit; Yellow regions: limbic circuit. **(B)** Schematic topography of the pallido-subthalamic afferents. Red regions: motor circuit; Green regions: exclusively associative circuit; Yellow regions: associative and limbic circuit. STh, Subthalamic Nucleus; SN, Substantia Nigra; GPi, Internal Globus Pallidus. GPe, External Globus Pallidus; Pu, Putamen; Th, thalamus; Ant. Dors Th, Anterior Dorsal nuclei of the Thalamus; Cau, Caudate nucleus; CC, Corpus Callosum; IC, Internal Capsule; Opt, Optic Tract; LF, Lenticular Fasciculus; AL, Ansa Lenticularis (for representation purposes, the topographical relationship between structures within the anteroposterior levels were simplified and depicted with the same schematic).

A more detailed description of the subthalamo-pallidal topography has been proposed by Joel and Weiner (1997) by integrating the findings of Nauta and Cole (1978); Carpenter and Jayaraman (1990), Shink et al. (1996) and Smith et al. (1990, 1995): the medial third of the rostral two-thirds of the STh projects mainly to the rostral GPe (associative circuit), to the ventral pallidum (limbic circuit) and to the rostral-ventromedial GPi (associative and limbic circuits); the lateral two thirds of the rostral two thirds of the STh project mainly to the central and caudal GPe and GPi; in particular, the dorsolateral two-thirds of this region project to the ventrolateral GPe and GPi (motor circuit), while the ventrolateral third projects to the dorsomedial third of the GPe and GPi (associative circuit). The caudal STh projects mainly to the ventrolateral GPe and GPi (thus converging on the motor circuit) apart of a small ventromedial portion projecting toward the associative circuit (dorsomedial GPe and GPi). This organization appears to be coherent with the hypothesis of the functional tripartition of the STh. Figure 5 shows a schematic representation of the aforementioned topography.

On both segments, the subthalamo-pallidal efferents terminate in the form of dense elongated bands that lie parallel to the medullary laminae (Parent and Hazrati, 1995). The target pallidal cells present an elongated dendritic domain parallel to the medullary laminae and are organized in rostrocaudally oriented cell layers, with each subthalamo-pallidal band contacting several pallidal cells. These bands are formed by thick non-varicose axons coursing in a caudorostral direction and giving rise to multiple thin and varicose axon collaterals forming a dense network around the dendrites and soma of pallidal neurons (Parent and Hazrati, 1995). This organization appears to be very similar to the vertically oriented terminal bands formed by striato-pallidal fibers, with the main difference being the distribution of the efferent arborizations: while the subthalamo-pallidal efferents present a quite uniform arborization throughout the whole extent of the pallidal complex, the striato-pallidal projections present a more heterogeneous arborization pattern and an opposite directionality. Furthermore, the proximal part of the striatal axons contacts the soma of the pallidal neurons while the terminal portion of the axon branches in order to entwine the dendrites of a more caudally located pallidal neuron; conversely, the subthalamic axons send numerous collaterals which entwine the dendrites and soma of several pallidal neurons, with the main axon continuing its caudorostral course (Parent and Hazrati, 1995). This indicates that the STh exerts a relatively widespread influence on the pallidal segments, contacting vast fields of pallidal neurons.

#### Pallido-Subthalamic Projections

##### Non-human Primate Tracing Studies

One of the main afferent pathways to the STh is represented by the pallido-subthalamic fibers, which originates exclusively from the GPe (Parent and Hazrati, 1995; Joel and Weiner, 1997, 2000; Sato et al., 2000a; Hamani et al., 2004). To date, no afferences from the GPi have been described; hence, the term pallido-subthalamic afferences refers to axons arising from the GPe. The majority of pallidal terminals within the STh exhibited numerous varicosities reminiscent of boutons en passant or boutons terminaux and displayed GAD and GABA immunoreactivity, forming synapses predominantly with proximal dendrites and less frequently with the soma and the distal dendrites (Parent and Hazrati, 1995; Sato et al., 2000a).

Rostral GPe (associative and supposedly limbic circuit) projects to the medial two-thirds of the rostral STh and to the central third of the middle STh, and to a lesser extent to the medial third of the central STh. Central GPe can be divided into two parts: (1) the dorsal two thirds, part of the associative circuit, project to the lateral two thirds of the central regions of the STh; (2) the ventral third of the GPe, part of the motor circuit, projects along with the whole caudal third of the GPe to the lateral and caudal regions of the STh. The ventral pallidum (part of the limbic circuit) appears to be reciprocally connected to the limbic STh. There also appears to be an inverse dorsoventral topography, with dorsal regions of the GPe projecting more ventrally, and ventral regions projecting more dorsally in the STh.

More recently, Karachi et al. (2005) studied the pallido-subthalamic pathway with particular regard to recent evidence on the functional subdivision of the GPe. According to the authors, the motor GPe is connected to the posterior and dorsal regions of the STh; the associative GPe is connected to the anterior central and posterior ventrolateral STh, while the Limbic GPe is connected with the anterior medioventral STh. These findings are coherent with the functional tripartite hypothesis of the STh and in accordance with previous literature.

Figure 5 evidences the aforementioned topography. Comparing the afferent projections to the efferent projections exposed in the previous paragraph, a correspondence between the GPe terminal field in the STh and the area of the nucleus projecting to the corresponding region of the GPi was evidenced, but appeared not to be absolute. Furthermore, the associative GPe appears to be reciprocally connected with the STh, but also projects to the dorsolateral regions of the central STh which are primarily connected to the motor pallidum. The pallido-subthalamic projection is thus considered as the essential component of the indirect pathway of the basal ganglia, conveying striatal information to the STh and GPi (Parent and Hazrati, 1995).

##### Human Histology and Tractography Studies

Lambert et al. (2012) evidenced a topographical organization of GPe connections with the STh: the posterior GPe is connected to the posterior STh (motor regions), while the middle GPe is connected to the anterior STh. This appears to be coherent with the functional subdivision of the GPe and with data deriving from non-human primate tracing studies. Pujol et al. (2017) explored the connectivity between the STh and the GP *in vivo* using fiber tracking. The fibers connecting the STh and the GPe crossed the anterior two-thirds of the posterior limb of the internal capsule and connected to the mediodorsal and medioventral aspects of the GPe. Connections between the STh and the GPi were found within the ventromedial aspect of the STh, while no topography of the projections on the GPi was reported. Plantinga et al. (2016) employed Ultra-high field MRI (7T) on a human *ex vivo* specimen to study the structural connectivity between the STh, the GP and the SN. The authors evidenced distinct connections with anteromedial aspect of the STh; these fibers circled around the internal capsule and connected to the anterior and inferior margins of the GPi, following a course compatible with the ansa lenticularis. The STh connections with the GPe, on the other hand, coursed either anteriorly around the internal capsule, in a bundle dorsal to the fibers connected to the GPi, or could be found running through the internal capsule and connected to the medial border of the GPe.

Recently, Alho et al. (2019) employed histological sections, ultra-high field MRI and connectome analysis to describe a bundle of fibers connecting the anterior and medial STh to the ventral GPi in humans. This bundle of fibers, known as ansa subthalamica, appears to be distinct from other bundles connecting the STh to the pallidal complex such as the ansa lenticularis, and constitutes a fundamental anatomical pathway for the limbic circuit of the basal ganglia. According to the authors this bundle could represent an interesting target for stereotactic surgery in psychiatric disorders, as it may play a crucial role within the fronto-striato-subthalamo-pallidal network engaged in goal-directed behaviors (Alho et al., 2019).

##### Further Perspective

While the connections between the GPe and STh have been extensively studied in non-human primates, data deriving from human subjects is still scarce. The connections between functional territories of the STh and functional territories of the GPe require more investigation in humans, and must be guided by the large amount of anatomical evidence deriving from non-human primate studies.

### Striatal Connections to the STh

#### Subthalamo-Striatal Projections

##### Non-human Primate Tracing Studies

Subthalamic projections to the Striatum, both Caudate nucleus and Putamen, are scarce compared to other subthalamic targets (Nauta and Cole, 1978; Smith and Parent, 1986; Parent and Smith, 1987; Sato et al., 2000b). According to Smith and Parent (1986; Parent and Smith, 1987), projections to the putamen arise mainly from the dorsolateral STh (part of the motor circuit), while projections to the caudate nucleus arise mainly from the ventromedial associative and limbic regions of the STh. According to Sato et al. (2000b), approximately 17% of the STh neurons labeled present a single axon projecting toward the striatum. However, the terminal arborizations of these labeled neurons could not be visualized in this study.

Subthalamo-striatal fibers are characterized by long, varicose axons with few collaterals, scattered throughout wide areas of both striatal components (Smith and Parent, 1986; Parent and Smith, 1987; Parent and Hazrati, 1995). According to Parent and Hazrati (1995), these projections likely exert an en passant type of excitatory influence on vast populations of striatal cells. No striato-subthalamical projections have been evidenced in non-human primates so far.

##### Human Fiber Tracking Studies

According to Lambert et al. (2012) the posterior putamen is connected to the motor STh, in particular the posterior parts of the nucleus, while the mid-inferior putamen is connected to the limbic STh, in particular the anterior regions of the nucleus.

##### Further Perspectives

Literature on STh connections with the Striatum in both non-human primates and humans appears scarce and requires further investigation. Considering the functional subdivision of the striatum and the morphofunctional difference between striosomes and matrix, it could be interesting to define if, and how, these territories are connected with the functional subdivisions of the STh.

### Nigral Connections With the STh

#### Subthalamo-Nigral Projections

##### Non-human Primate Studies

The substantia nigra represents one of the main targets of the STh (Carpenter et al., 1981b; Parent and Smith, 1987; Smith et al., 1990; François et al., 2000). These fibers enter the substantia nigra pars reticulata (SNr) mainly by coursing through the cerebral peduncle, arborizing along the basis of the SNr and forming several distinct terminal plexuses (Carpenter et al., 1981b; Parent and Hazrati, 1995). Even though most fibers terminate at the level of the reticulate part, some fibers ascend along the dopaminergic cell columns of the substantia nigra pars compacta (SNc), thus influencing both dopaminergic and non-dopaminergic cells (Smith et al., 1990). The subthalamo-nigral pathway appears to arise from neurons mainly in the ventromedial regions of the STh, displaying an approximate mediolateral topography (Smith et al., 1990). At the level of the substantia nigra, these fibers form several terminal fields consisting of networks of axon collaterals showcasing both en passant and terminal boutons (Smith et al., 1990); prominent perisomatic arborization has been highlighted, but appears to be inconsistent throughout different studies. These terminals showcase certain similarities to the subthalamo-pallidal terminals, suggesting that both projections arise from the same neuronal population (Parent and Hazrati, 1995).

#### Nigro-Subthalamical Projections

The nigro-subthalamical pathway appears to be less prominent in primates than in rodents (Parent and Hazrati, 1995). While the nucleus appears to be surrounded by several dopaminergic fiber systems, only few appear to contact its neurons by entering the mediodorsal regions. According to tracing studies in rodents, the nigro-subthalamical projection arises mainly from the substantia nigra pars compacta, while the STh projects mainly to the pars reticulata (Brown et al., 1979).

In non-human primates, François et al. (2000) evidenced extensive projections arising from dopaminergic areas A8, A9, and A10 in the mesencephalon. Through anterograde tracing, the authors confirmed projections arising from the mesencephalon and targeting the STh. The dopaminergic nature of these afferences was confirmed through positivity for tyrosine hydroxylase. In particular, the labeled axons originating from the mediodorsal part of area A9 project to the anteriomedial STh, while the fibers originating from area A8 project to the whole extent of the STh.

The dopaminergic fibers arising from the substantia nigra appear to be characterized by numerous axon varicosities; some axons branch extensively within the STh in order to contact numerous target neurons, while other axons selectively target very few neurons within the structure (Lavoie et al., 1989; François et al., 2000; Hamani et al., 2004).

##### Human Tractography Studies

Connections between the STh and the SN were reported in a post-mortem human specimen by Plantinga et al. (2016) using DWI. The authors evidenced connections between the inferolateral border of the STh and the superior border of the SN. The connection with the SNc is located more anteriorly within the STh compared to the connections with the SNr. According to the authors, the connection with the SNc appears to be significantly larger than the connection with the SNr.

##### Further Perspectives

Few studies have addressed the structural connectivity between the STh and the substantia nigra in non-human primates and in humans. Aspects such as the precise termination of SN projections to the STh remain to be clearly determined. From a functional perspective, the dopaminergic innervation of the STh may play a fundamental role in the regulation of STh afferences, such as corticosubthalamic fibers (Mathai and Smith, 2011). From an anatomical perspective, the exact identification and subcellular localization of dopaminergic synapses within the human STh could significantly improve our understanding of the dopaminergic modulation of the STh. Considering the topographical vicinity between the STh and the SN, *ex vivo* tracing methods could be employed to study the connections between the SN and the STh without incurring in significant technical difficulties related to tracer transport over long distances (i.e., from the cortex to subcortical structures) within *ex vivo* human brain tissue. While this information does not resolve most of the questions posed by Mathai and Smith (2011) regarding the physiological and pathological implications of dopaminergic modulation of STh afferences, it could represent a starting point for the morphofunctional characterization of extrastriatal dopaminergic innervations in humans.

### Thalamic Connections With the STh

#### Non-human Primate Tracing Studies

The thalamo-subthalamic pathway arises mainly from the parafascicular nucleus of the thalamus and from the centromedian nuclei (Sadikot et al., 1992; Tandé et al., 2006). The centromedian nucleus appears to project mainly to the motor division of the STh, in particular to the dorsolateral regions of the nucleus. The parafascicular nucleus innervates mainly the medial and rostral regions of the STh, constituting the limbic/associative territories of the nucleus. The axon terminals of this pathway appear to contact mainly the dendrites of the STh cells, displaying glutamatergic immunoreactivity (Hamani et al., 2004).

More recently, bidirectional connections with the ventral anterior and ventral lateral thalamus have been described (Rico et al., 2010). These fibers connect mainly the medial STh to the aforementioned thalamic nuclei, playing a role in both motor and associative control.

### Cerebellar Connections With the STh

Even though indirect connections arising from and terminating on the STh were not considered within this review, the recent discovery and the morphofunctional relevance of indirect connections between the STh and the Cerebellum could represent an important stimulus to functional and connectivity research, along with their clinical implications, within the network of basal ganglia circuits, and will therefore briefly discussed.

#### Non-human Primate Tracing Studies

Recently, indirect connections between the STh and the cerebellum have been identified by Bostan et al. (2010). Through the injection of rabies virus (retrograde tracer) in the cerebellum of non-human primates, the authors identified a disynaptic pathway connecting the STh to the pontine nuclei, which in turn projected toward the controlateral cerebellar cortex. In particular, injections within the Crus IIp of the cerebellum displayed numerous labeled neurons within the rostral and ventromedial STh, while injections within the lobule VIIb displayed numerous labeled neurons within the caudal STh. According to the authors, modulation of the subthalamo-cerebellar pathway could be provided by the nucleus reticularis tegmenti pontis and other pontine nuclei, which are also structurally connected to the STh (Giolli et al., 2001).

#### Human Fiber Tracking Studies

Based on Bostan et al.’s (2010) results, Pelzer et al. (2013) identified connections between the STh and the cerebellar cortex in living humans through DTI. According to the authors, the STh was connected to both regions identified by Bostan et al. (2010), but appeared to also be connected to lobule VIII and IX. These fibers crossed the midline at the level of the pons and entered the cerebellum passing through the middle cerebellar peduncle.

Milardi et al. (2016) extensively examined the connections between the basal ganglia and the cerebellum, confirming and expanding the findings of previous authors. However, unlike Pelzer et al. (2013), the authors identified an ipsilateral connection between the STh and the cerebellar cortex.

Wang et al. (2020) employed high definition fiber tractography to identify cortico-subthalamo-cerebellar connections. According to the authors, fibers connecting the STh and the cerebellum pass through the cerebral peduncle along the course of the cortico-cerebellar fibers. At the level of the pons they connect in two opposite directions to join the middle cerebellar pedicle on both sides. Most fibers connected bilaterally to Crus I in the cerebellum, while the remaining fibers crossed the midline and connected to Crus II. Interestingly, the authors evidenced a positive correlation between the numbers of fibers connecting mesial BA8 to the STh and the number of fibers connecting the STh with Crus I. Since the hyperdirect pathway between BA8 and the STh seems to be involved in decision making (Wang et al., 2020), the fibers connecting the STh to Crus I and II could represent a continuation of such pathway and also contribute to higher cognitive functions.

#### Further Perspectives

While human fiber tracking studies provide compelling information on the subthalamo-cerebellar circuitry, more data deriving from non-human primate tracing studies is required to further characterize these connections. In fact, it must be considered that Bostan et al. (2010) tracing study is based on the observation of only two subjects and two main injection sites.

The identification of second order neurons and mediating nuclei, along with the topographical organization of subthalamo-cerebellar projections represent aspects that require further clarification: is there a topographically organized projection arising from the motor areas of the STh that maintains the somatotopic representation of cortical afferences? What is the role of deep cerebellar nuclei, such as the dentate nucleus, in the subthalamo-cerebellar pathway? Hopefully, more non-human primate tracing studies will provide information on these aspects.

### Brainstem Connections to the STh

#### Non-human Primate Tracing Studies

The STh appears to be connected to several brainstem nuclei, such as the pedunculopontine tegmental nucleus (PPT), the ventral tegmental area of the mesencephalon, the periacqueductal gray and the dorsal raphe nucleus (Nauta and Cole, 1978; Carpenter et al., 1981b; Parent and Smith, 1987; Smith et al., 1990; Lavoie and Parent, 1994a, b, c). The PPT projects conspicuously to the whole extent of the STh, representing the main cholinergic input to the nucleus (Lavoie and Parent, 1994c). These fibers appear to modulate the activity of neurons in the STh and other basal ganglia structures. A minor pathway arising from the STh and directed toward the PPT has also been identified, which is thought to relay basal ganglia information to the lower brainstem and spinal cord via the mesencephalic locomotor region (Parent and Hazrati, 1995), in particular through the activation of the nucleus reticular gigantocellularis, which regulates the activity of spinal interneurons through the reticulospinal tract (Pahapill and Lozano, 2000; Hamani et al., 2004). Thus, the circuit appears to be involved in the facilitation of locomotion and in the regulation of cardiorespiratory activity during motor exercise (Eldridge et al., 1985). According to Lavoie and Parent (1994c), the terminals of the PPT-STh projection form contacts with the soma and proximal dendrites of STh neurons; however, these information seem to derive from rodent and feline studies (Morizumii et al., 1987). Information on these terminals in non-human primates seems to be lacking.

The dorsal raphe nucleus appears to consistently innervate the STh through widespread 5-HT immunoreactive fibers, even though their physiological activity remains controversial (Rinvik et al., 1979; Carpenter et al., 1981b; Mori et al., 1985).

Several other nuclei projecting to the STh have been identified, even though the organization and the functional role of these projections remains unclear; these include: the reticular nucleus of the thalamus, the hypothalamus, the amygdaloid nuclei, the locus coeruleus, the zona incerta and the parabrachial nuclei (Rinvik et al., 1979; Canteras et al., 1990).

#### Human Fiber Tracking Studies

Through DTI, Muthusamy et al. (2007) and Aravamuthan et al. (2007) were the first to identify the connection between the PPT and the STh in humans. In particular, the two structures appeared to be connected at the level of the medial, posterior and superior eight of the PPT and at the level of the medial and inferior STh (Aravamuthan et al., 2007). Again, results deriving from diffusion based approaches should be interpreted with care: even though the connections evidenced largely match those found in animal studies, all non-human primate axon tracing studies known to us identify projections arising from the PPT with diffuse terminations and extensive branching throughout the whole extent of the STh, and do not describe an inferomedial termination of afferences (Nauta and Cole, 1978; Carpenter et al., 1981b; Parent and Smith, 1987; Smith et al., 1990; Lavoie and Parent, 1994a, b, c). Interestingly, Lavoie and Parent (1994c) evidenced that PPT fibers enter the STh from its medial tip and dorsal surface and arborize profusely and uniformly throughout the nucleus; considering the technical limitations of DTI, it could be speculated that Aravamuthan et al.’s (2007) study reconstructed PPT projections to the STh entering the nucleus from its medial tip, in accordance to animal studies, without being able to reconstruct the arborization of these fibers within the structure. More evidence from human fiber tracking studies, possibly with higher spatial resolution, could help to clarify this aspect.

### Synaptic Contacts of the Major Afferents to the Subthalamic Nucleus

Table 3 shows the distribution and cellular localization of synaptic contacts at the level of STh cells. Fibers originating from the motor cortex (in particular area M1 and PMC), centromedian and parafascicular nuclei of the thalamus, substantia nigra pars compacta, pedunculopontine tegmental nucleus and dorsal raphe nucleus contact STh neurons mainly at the level of distal dendrites (Rinvik et al., 1979; Carpenter et al., 1981b; Mori et al., 1985; Morizumii et al., 1987; Smith et al., 1990; François et al., 2000; Tandé et al., 2006; Rico et al., 2010; Coudé et al., 2018), whilst pallidal fibers contact mainly the soma and proximal dendrites of STh cells (Parent and Hazrati, 1995; Sato et al., 2000a).

**TABLE 3:** Localization of subcellular afferences to monkey STh.

Afferent structure	Principal neurotransmitter	Localization of main synaptic contacts	References
Motor cortex	Glutamate	Distal dendrites and spines	Coudé et al., 2018
GPe	GABA	Cell body and proximal dendrites	Parent and Hazrati, 1995; Sato et al., 2000a
Thalamus	Glutamate	Distal dendrites	Tandé et al., 2006; Rico et al., 2010
SNc	Dopamine	Dendrites	Smith et al., 1990; Parent and Hazrati, 1995; François et al., 2000
PPT (Tegmentum)	ACh	Distal dendrites and soma	Rodent and feline studies (Morizumii et al., 1987); confirmation required from non-human primate studies.
Dorsal Raphe	5-HT	Distal dendrites	Rinvik et al., 1979; Carpenter et al., 1981b; Mori et al., 1985

## Funcional Segregation or Functional Convergence? Anatomical Evidence of STh Open and Closed-Loop Circuits

The previous paragraphs highlighted the intricate network of connections directed to and originating from the STh. Given the tripartite division of the striatum, pallidum and STh, the parallel segregated principle predicts the existence of indirect pathways connecting functionally corresponding subregions of the striatum, GPe, GPi and STh. In particular, it has been evidenced that efferents originating from the GPe contact STh neurons projecting to GPi and SNr, thus providing an anatomical support for the existence of an indirect pathway (Joel and Weiner, 1997). Initially the original hypothesis was rejected by Parent and Hazrati (1995) due to lack of firm anatomical evidence for the existence of the indirect pathway, as previous studies were unable to confirm GPe connections to the STh territory containing neurons projecting toward the GPi/SNr. According to the authors, an indirect pathway connecting the GPe to the output structures of the basal ganglia (i.e., GPi and SNr) required either direct connections between the GPe and the aforementioned nuclei, or an indirect GPe-STh-GPi/SNr projection, which in turn relied on the existence of a link between the dorsolateral STh (receiving GPe input) and the ventromedial STh (giving rise to GPi/SNr projections) (Parent and Hazrati, 1995). Supporting data was subsequently provided by Shink et al. (1996), identifying the connection between GPe fibers and STh neurons projecting to the GPi through electron microscopy.

However, the functional segregation initially proposed appears to be only partially confirmed: segregation seems to be maintained with regard to the associative subregions of the striatum (associative regions being contacted exclusively by other associative regions), but not at the level of the motor circuits (motor regions being contacted by other functional division, in particular the associative ones).

Two possible hypotheses arise:

1. The associative and motor circuit project to the same neurons of the motor STh, which integrates the information originating from both circuits and redirects them to the motor GPi.
2. The associative circuit and the motor circuit project to different subpopulations of neurons within the STh. These subpopulation would differ only for the afferences received, whilst projecting on the same neurons at the level of the motor GPi.

However, regardless of whether the projections from the motor and associative GPe remain segregated in the motor STh or converge on the same neurons, information is still transferred from the associative striatum through associative GPe and motor STh to the motor GPi (Joel and Weiner, 1997). The consequences of this anatomical organization will be discussed further.

According to Joel and Weiner’s hypothesis (1997), two types of indirect pathways can be identified in regard to the topographical organization of STh afferent and efferent projections:

1. Closed indirect pathway: this pathway terminates in the same GPi/SNr subregion as the direct pathway arising from the corresponding striatal subregion. This leads to the connection of functionally related regions of the striatum, pallidal complex and STh according to the parallel segregation scheme. The closed indirect pathway contributes to the processing of information within the basal ganglia-thalamocortical circuits.
2. Open indirect pathway: this pathway terminates in a different subregion of the GPi/SNr than the direct pathway, thus connecting functionally non-corresponding subregions of the striatum, pallidal complex and STh. The open indirect pathway contributes to the connection between circuits of the basal ganglia.

Thus, three possible closed pathways can be hypothesized, each one connecting the corresponding functional regions of the striatum, pallidal complex, STh and SNr. On the other hand, among the open indirect pathways it is possible to identify the one linking the associative striatum to the motor GPi passing through the associative GPe and motor STh (one further open indirect pathway is the one connecting the associative regions to the ventral pallidum and the limbic circuit; for more information, consult Joel and Weiner, 1997).

Empirical evidence for the open versus closed loop circuits hypothesis in non-human primates and humans remains controversial. Kelly and Strick (2004) employed rabies virus retrograde tracing to define closed and open loop circuits within the primate cortico-basal ganglia-thalamo-cortical circuits. Even though the authors evidenced mostly closed-loop circuits between motor and limbic areas, an open-loop circuit connecting (indirectly) the ventral putamen to M1 has also been identified. The ventral putamen belongs to the limbic circuit of the basal ganglia (Parent and Hazrati, 1995), and seems to be connected to limbic structures such as the amygdala (Parent and Hazrati, 1995; Joel and Weiner, 1997; Kelly and Strick, 2004). According to the authors, this connection could represent the anatomical demonstration in primates for an open-loop circuit between the limbic circuit and the motor circuit of the basal ganglia.

More recently, a study on rodents (Aoki et al., 2019) has provided some interesting insight on the anatomo-functional organization of closed and open loop circuits within the rat basal ganglia. By combining genetic and viral approaches, the authors mapped the limbic and motor circuits between the cortex, the basal ganglia and the thalamus in rodents. Despite evidencing largely closed loops within each functional domain, the authors discovered an unidirectional influence of the limbic over the motor loop via ventral striatum-substantia nigra (SNr)-motor thalamus circuitry; furthermore, activity within the ventral striatum of the rat seems to modulate the activity of the primary motor cortex. These results appear to be in line with Kelly and Strick’s (2004) findings in non-human primates. Interestingly, this pathway seems to form synapses with the SNr, rather than the GPi. Kelly and Strick (2004) did not report any retrogradely labeled neurons within the SNr, but only in the GPi. Therefore, it could be speculated that the non-human primate open loop circuit connecting the ventral putamen to the motor cortex passes through the GPi, rather than the SNr. According to Hardman et al. (2002), the SNr plays a much more important role as an output structure of the basal ganglia in rodents compared to non-human primates, while the opposite seems to be true for the GPi.

Considering the increasing importance of the STh in processing different types of information through phylogenesis (Hardman et al., 2002), the definition of the STh’s role in open versus closed loop circuits could represent an important aspect regarding information processing within the human basal ganglia.

## Conclusion and Future Perspectives

Even though the STh represents a topic of interest in current neuroscience research, most anatomical data available derives from histological and tracing studies in non-human primates. Data on humans is limited to very few studies employing mostly MRI in combination with unbiased stereology applied to histological sections. According to our perspective, it is possible to identify three main points of interest within human STh research which require further and detailed investigation: connectivity, cytoarchitectural and chemoarchitectural organization, and functional subdivision.

### Connectivity

Currently, the connectivity of the STh has been investigated in non-human primates through *in vivo* tracing studies, and in humans in both *in vivo* and *ex vivo* specimen through MRI, specifically through DTI and DWI. Conversely, the connectivity of the human STh has never been investigated through post-mortem tracing methods, such as Carbocyanine tracing or Neuro Vue Dyes. These tracers allow for the anterograde and retrograde tracing of axons even in formalin fixed human specimens (Heilingoetter and Jensen, 2017), and could be employed to study the connectivity between the STh and closely related structures, such as the substantia nigra, the globus pallidus, and the nucleus of the zona incerta. For long distance tracing, complications may arise due to the very long incubation times required (from several months to years) and due to technical difficulties with the cryostat-sectioning of macrosections. Even though these techniques may present inherent limitations, such as long incubation periods and possible tracer diffusion during sectioning, they also appear to be significantly more accurate than *in vivo* tracing studies (Heilingoetter and Jensen, 2017); furthermore, given the increasingly important role of the STh through phylogenesis (Hardman et al., 2002), these tracing techniques could identify a different organization of afferent and efferent projections in humans compared to monkeys, whilst also expanding on the topography of STh connections.

Post-mortem tracing techniques can be used in conjunction with ultra high field MRI, DTI and DWI of *ex vivo* specimens to confirm the anatomo-radiological findings and to expand on several aspects of connectivity which are still unclear, such as the topography of projections, eventual subpopulations of target cells and the type of synapse formed (e.g., axo-axonic, axo-somatic, or axo-dendritic synapses).

### Cytoarchitectonics and Chemoarchitectonics

While the cellular morphology and different cellular populations have been identified in rodents, cats and primates, there are no conclusive studies in humans. The morphology of human subthalamic neurons can be studied through Golgi silver impregnation techniques, such as the Golgi-Cox and Golgi-De Bubenaite methods, even though formalin fixed human tissue generally leads to artifact formation and incomplete precipitation of metallic silver, if not appropriately pre-treated. On the other hand, fresh tissue can be impregnated using osmic acid as a fixative (Golgi-Cox variation), after isolating the STh in a freshly cut hemisphere. Otherwise, carbocyanines and Neuro Vue dyes can also be employed to evidence the morphology of single neurons and, unlike silver impregnation techniques, also allow for the visualization of synaptic contacts in traced neurons.

A general overview of the axonal and dendritic network within the STh can be further investigated through the aid of the Cajal-De Castro photographic silver techniques, allowing for the impregnation not only of formalin fixed bulks of tissue, but also of paraffin embedded sections.

Immunohistochemistry should be employed to characterize the distribution of different receptors or molecules of interest; it must be noted, however, that the quality of Immunohistochemistry depends on the method and time of fixation: specimen fixed for longer periods (>1 year) in formalin yield generally worse results than fresh and appropriately fixed specimen. The definition of neuronal populations expressing specific proteins or receptors, as well as their distribution within the structure, should be investigated through the aid of unbiased stereology in both health and disease, with particular regard to movement disorders and α-sinucleopathies. The chemoreceptorial characterization of STh neurons could help define fundamental functional aspects of the structure.

Hence, both cytoarchitectonics and chemoarchitectonics could provide evidence for different subpopulations of neurons within the STh, receiving and processing different types of informations, with particular regard to the functional subdivision hypothesis.

### Functional Subdivision

This aspect remains one of the most crucial in STh research. The identification of functional subdivisions within the nucleus, and their clear definition in terms of topography, connectivity, cell population, and receptor and protein expression could provide novel insights on how different types of information are processed within the basal ganglia. Furthermore, considering the fundamental role played by the STh in DBS, the identification of functional divisions of the structure which are both safe to stimulate and easy to target could significantly improve treatment outcome.

## Author Contributions

AA conceived the review. AE drafted the manuscript and designed the figures. AE, RD, AP, and VM revised the manuscript. All authors contributed to the final manuscript.

## Conflict of Interest

The authors declare that the research was conducted in the absence of any commercial or financial relationships that could be construed as a potential conflict of interest.

## Footnotes

1 The abbreviation “STh,” as seen in G. Paxinos’s texts, is preferred over the more widely used “STN,” as it may be confused for an abbreviation of Solitary Tract Nucleus.
